# Impacts of Fungal Infection and Mycotoxin Contamination on Nutritional Quality and Safety of Protein-Rich Pulses

**DOI:** 10.14252/foodsafetyfscj.D-25-00005

**Published:** 2026-09-25

**Authors:** Elizabeth M. Wyman, Emily Branstad-Spates, Kanniah Rajasekaran, Matthew D. Lebar

**Affiliations:** Food and Feed Safety Research Unit, Southern Regional Research Center, United States Department of Agriculture, Agricultural Research Service, New Orleans, LA 70124, USA

**Keywords:** alternative proteins, plant-based proteins, pathogenic fungi, *Aspergillus*, *Fusarium*, *Penicillium*

## Abstract

Plant-based protein sources are increasingly incorporated into human diets as the search for more sustainable and nourishing food sources arises. Numerous fungal plant pathogens that impact pulses and plant-based proteins, namely *Aspergillus flavus*, *Fusarium oxysporum* and certain *Penicillium* species, have been studied widely in corn, peanut and cotton previously. We review reports of these fungal genera and their infections in pulses, as well as other pathogenic and mycotoxigenic fungi reported to infect and contaminate pulses. Overall, plant-based protein sources have been understudied in the United States (US) and abroad. This review captures current research (through 2025) to reveal how pulse crops, an emerging source of plant-based proteins, are impacted nutritionally by fungal infections and their subsequent mycotoxin contamination. The major findings are 1) that fungal infections tend to decrease nutritional composition of pulse seeds and 2) that there are consistent gaps in current pulse research that need to be addressed.

## 1. Introduction

Historically, animal agriculture, including the production of meat, eggs, and dairy, has been the primary source of protein in human diets. Currently 75% of agricultural land is used for raising and feeding livestock, while supplying a mere 1/3^rd^ of the world’s protein^1^^)^. However, the depletion of natural resources due to the increasing global population has become a critical issue worldwide^2^^)^. So, as the human population grows, there’s a rising demand for healthy, sustainable, and nourishing food sources that do not further strain global resources. To that end, plant-based proteins have emerged as non-traditional sources of protein that can supplement conventional animal-derived proteins^3^^)^. These plant-based proteins come from a variety of sources, including pulses (namely soybeans, peas, lentils, chickpeas, beans, and others). Pulses are seed products of the plants belonging to the family *Fabaceae* or *Leguminosae* and are widely considered grain crops. A recent review found that as pulses have been incorporated into various food products, it has created a more resilient, economical, and nutritious food system^4^^)^.

The plant-based protein industry is expanding rapidly and is projected to reach $27 billion by 2030^3^^)^. Plant-based proteins are gaining popularity due to their potential environmental and health benefits. Many pulses are considered excellent sources of protein because they have >20% daily reference value (DRV) of protein as compared to “good sources” of protein which have 10-19% DRV, based on FDA food labeling standards^5^^,^^6^^,^^7^^)^. Though pulses often lack certain essential amino acids, making them “incomplete proteins.” While high in some amino acids including lysine, pulses are deficient in methionine, cysteine, and tryptophan^8^^)^. To overcome these deficiencies, it is beneficial to combine pulses with cereals to ensure a more well-rounded amino acid profile is consumed.

Despite these limitations, the cost of producing pulses is significantly less than animal agriculture due to reduced water usage and lower greenhouse gas emissions^9^^,^^10^^)^. Additionally, pulses are highly productive per unit area, especially when grown in rotation with other crops. These food sources do face challenges such as susceptibility to diseases and pests, including fungal and bacterial diseases, nematodes, and insect damage. However, they offer a lower carbon footprint, improved soil health, cost-effectiveness, and versatility for a growing population^2^^,^^8^^)^. Pulses also offer several health advantages when included in human diets, such as improved cardiovascular health, better metabolism, obesity prevention, reduced cholesterol, and enhanced gut microbiome health due to their rich nutrient profile, including protein, fiber, vitamins, and minerals^11^^,^^12^^,^^13^^,^^14^^)^. Overall, as pulses have been incorporated into various food products, the food system has become more resilient, economical, and nutritious^4^^)^. Thus, ensuring the safety of plant-based proteins is crucial to meeting the growing consumer demand.

**Fig. 1.**
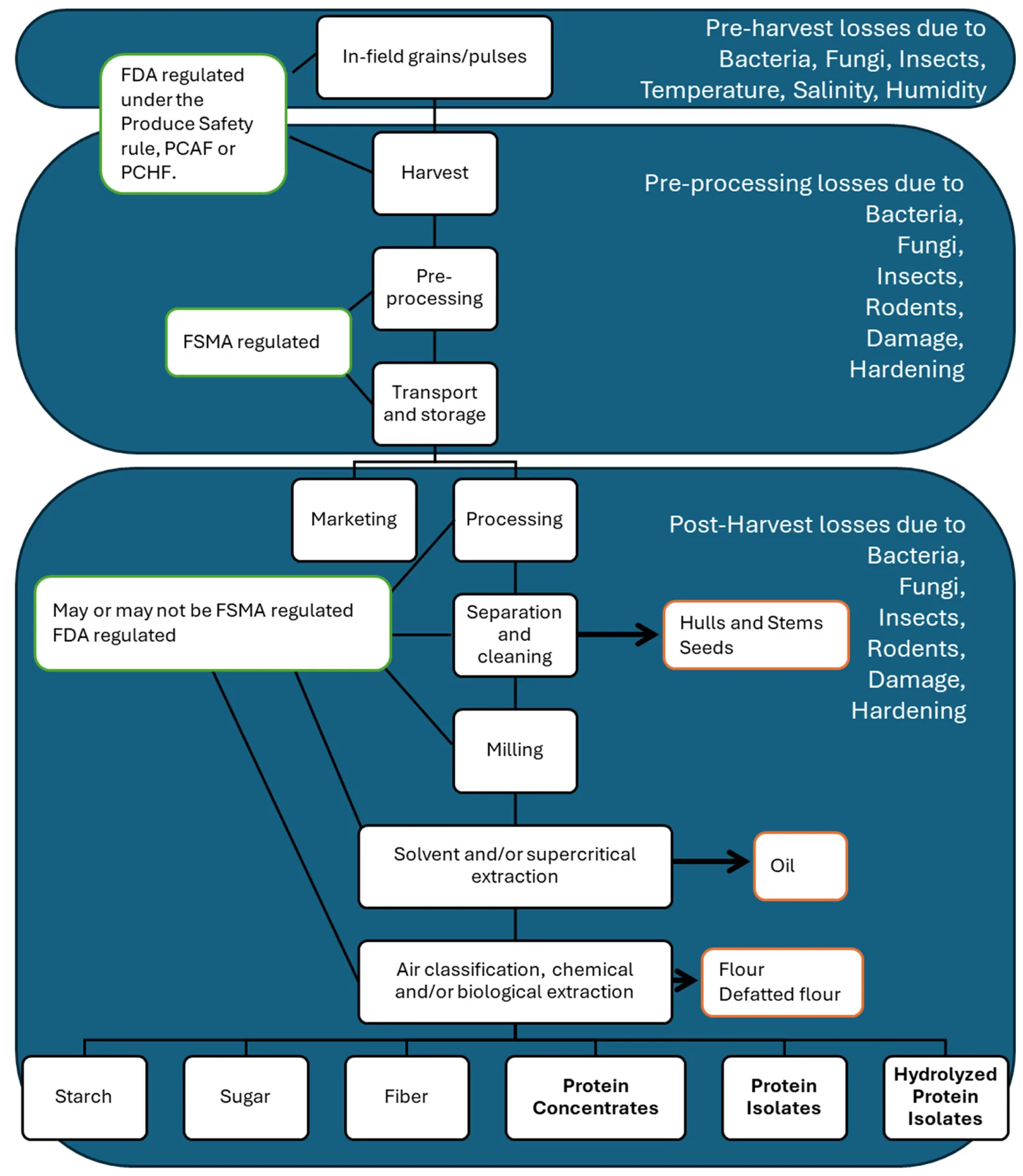
**The United States (US) Pulse Supply Chain.** Pulses are vulnerable to pre- and post-harvest contamination by many factors along the supply chain, including mycotoxigenic fungi, and only some of these areas are regulated. FSMA, Food Safety Modernization Act; FDA, Food and Drug Administration; PCAF, Preventive Controls for Food for Animals; PCHF, Preventive Controls for Human Food^1^^,^^177^^,^^178^^,^^179^^)^.

Mycotoxins, toxic secondary metabolites produced by fungi, can pose serious threats to human and animal health, causing acute and chronic conditions such as carcinogenesis, immunosuppression, and even potential death^15^^)^. Contamination of plant-based proteins by fungi, including mycotoxigenic fungi, is a significant concern and can occur at any stage in the supply chain, from field to storage (Fig. 1)^16^^,^^17^^)^. Currently, the estimated loss of pulses due to fungal contamination is up to 14% annually, although this varies widely depending on the year, type of plant-based protein, and regional differences^18^^)^.

There is limited research on the susceptibility of plant-based proteins to mycotoxigenic fungi compared to well-studied crops such as corn, peanuts, and cottonseed. Acuña-Gutiérrez et al. (2022)^16^^)^ provided a review on mycotoxins in pulses, discussing climatic conditions related to fungal colonization, mycotoxin accumulation, mechanisms of accumulation, and the physiological effects in seeds and seedlings. And it is worth noting that some pulses seem more resistant to aflatoxin and fungal colonization compared to corn^19^^)^. However, nutritional changes in plant-based proteins when infected with fungi, specifically mycotoxigenic fungi, need further exploration to ensure food and feed safety. Therefore, this review aims to describe current standards of food and feed safety in pulses and published information regarding the potential effects of mycotoxigenic fungi on pulses from a nutritional and food safety standpoint.

## 2. Fungal and Mycotoxin Contamination in Food Products Including Pulses

Mycotoxin contamination in pulses is a major food and feed safety concern globally. In a recent review, Kaur et al. (2025) emphasized the regulatory and safety needs of new pulse products as they become manufactured, traded, and consumed globally^20^^)^. In brief, the USDA-FSIS and FDA are working together to regulate plant-based proteins and their subsequent food products, like cultured meats, to be generally recognized as safe (GRAS)^20^^)^. Mycotoxins, produced by fungi like *Fusarium*, *Aspergillus*, *Penicillium*, and other species can infect pulses during growth, harvest, and storage. Among the various mycotoxins, aflatoxins (AFs) and fumonisins (FUMs) are particularly harmful, posing serious health risks such as carcinogenicity, neurotoxicity, and immunosuppression^21^^)^. Effective management strategies, including stringent agricultural practices, timely harvesting, adequate drying, and proper storage conditions, are crucial in minimizing contamination in pulses^22^^)^. Additionally, advances in detection technologies and breeding resistant crop varieties are critical areas of ongoing research that aim to safeguard public health and ensure the safe inclusion of pulses in the food supply chain.

Described below are mycotoxigenic fungi known to infect and contaminate pulse crops. Pulses have been previously referred to as “grain legumes,”^23^^)^ so we highlight mycotoxin action levels and limits set for cereals, nuts and grains to compare the differences across food products. To our knowledge only China specifically names “beans” in their reports. No other country routinely tests or regulates legumes for mycotoxin contamination, currently. We summarize and consolidate these action levels across many countries to serve as groundwork for future guidance to enhance the safety and quality of pulses and protein-rich products derived from pulses.

### 2.1 *Aspergillus* spp.

**Fig. 2.**
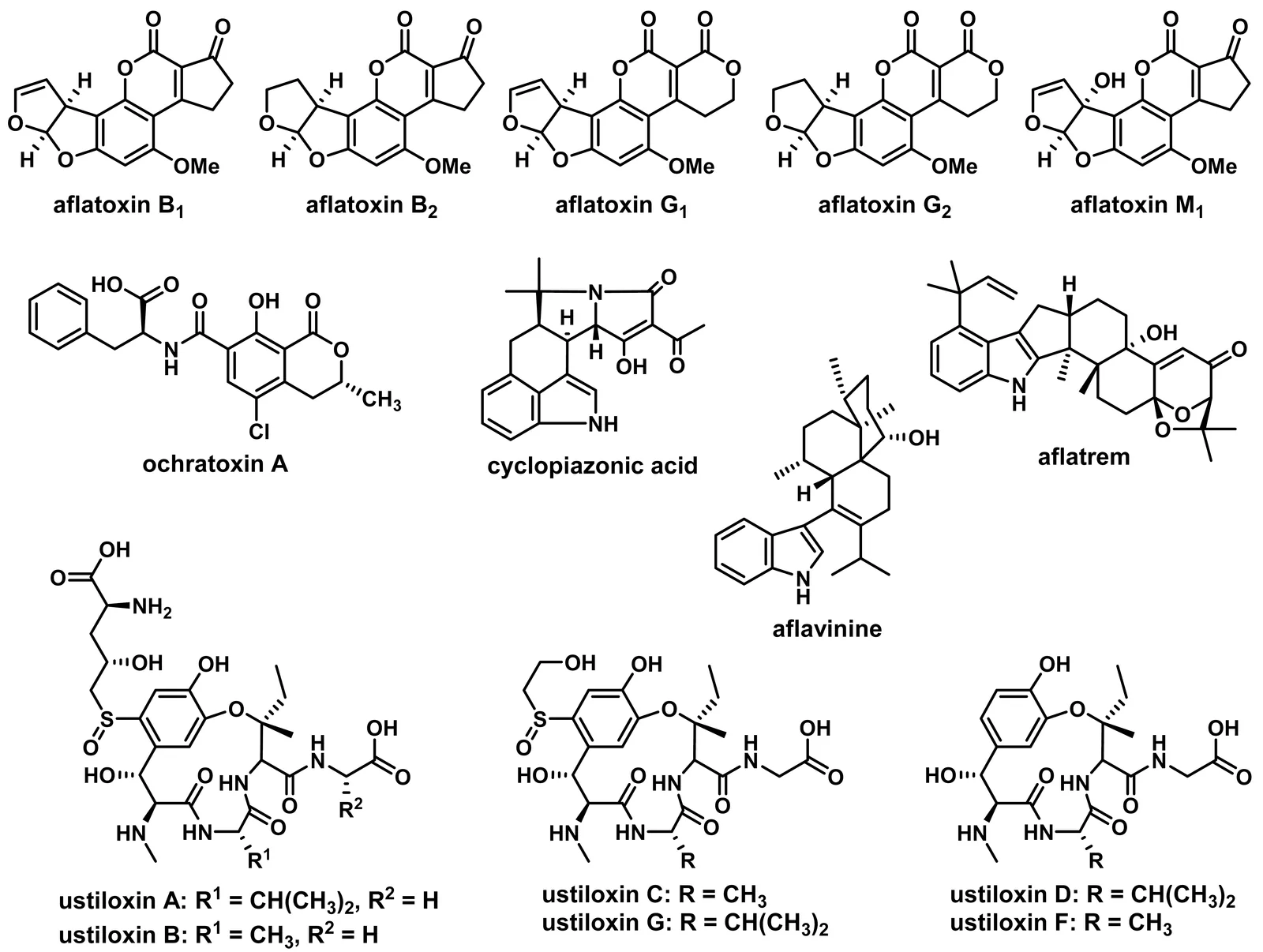
**Molecular structures for *Aspergillus* spp. toxins.** There are many major toxins produced by *Aspergillus* spp., including aflatoxin B_1_, B_2_, G_1_, G_2_, M_1_, ochratoxin A, ustiloxin A, B, C, D, G, and F, cyclopiazonic acid, aflatrem, and aflavinine.

Species of the genus *Aspergillus* are known to produce several mycotoxins that can pose significant health risks to both humans and animals^24^^)^. Common mycotoxins produced by *Aspergillus* spp., particularly *Aspergillus flavus* and *Aspergillus parasiticus*, include aflatoxins (AFs) B_1_, B_2_, G_1_, G_2_, M_1_ (the hydroxylated metabolite of AFB_1_ in milk), ochratoxin A (OTA), ustiloxins, patulin, cyclopiazonic acid (CPA), aflavinine and aflatrem (**Fig. 2**)^24^^)^. These mycotoxins can contaminate a wide variety of food products including cereals, nuts, spices, dried fruits, and coffee beans, and are especially prevalent in warm, humid conditions^25^^)^. To date, AF is the most studied mycotoxin in pulses^16^^)^.

#### 2.1.1 Aflatoxins (AFs)

Aflatoxins (AFs), toxic secondary metabolites produced by *A. flavus*, *A. parasiticus*, and related *Aspergilli*, contaminate a variety of food and feed crops globally, including well-studied crops such as corn, cottonseeds, peanuts, and tree nuts pre- and post-harvest^26^^)^. There are four major AFs (B_1_, B_2_, G_1_, and G_2_) and they are considered to be the mycotoxins of greatest concern from a global perspective^27^^)^. AFB_1_, the most potent natural secondary metabolite, is known for its strong carcinogenic, hepatotoxic, mutagenic, and teratogenic properties^28^^)^. The International Agency for Research on Cancer (IARC) has established AFB_1_ as a Group 1 carcinogen, and chronic exposure can lead to liver cancer, immunosuppression, and growth impairment^29^^)^. Additionally, AFB_1_ can be bio-transformed to the hydroxylated metabolite AFM_1_ excreted in milk and other dairy products^30^^)^.

AF is the most heavily regulated mycotoxin globally. The FDA has set action levels for AF in food and feed, and the set level for Brazil nuts, peanuts, pistachio nuts and other foods intended for human consumption is 20 ppb (µg/kg)^31^^)^. For animal feed, corn, peanut, and cottonseed meal intended for finishing beef cattle, swine, or poultry regardless of age and breeding status, the AF action level is set to 300 ppb (µg/kg). Corn and peanut products intended for finishing swine have an action level of 200 ppb (µg/kg), whereas corn and peanut products intended for breeding stock are set at 100 ppb (µg/kg). Lastly, AFM_1_ has an action level of 0.5 ppb (µg/L) in milk and milk byproducts^31^^)^. The European Union (EU) has established maximum levels (ML) for AFB_1_ at 2 ppb (µg/kg) in cereals and processed cereals for human consumption, and 0.05 for AFM_1_ in milk^32^^)^. China has established limits at 20 ppb (µg/kg) for total AFs in peanuts and maize, 5 ppb (µg/kg) for AFB _1_ in wheat, barley, or other grains and fermented bean products, and similar limits as the US for milk^33^^)^. Japan has set a regulatory level of 10 ppb (µg/kg) for total AF in all food items^34^^)^. The World Health Organization (WHO) and Food and Agricultural Organization (FAO) created a Joint FAO/WHO Expert Committee on Food Additives (JECFA), and JECFA has not set a provisional maximum tolerable daily intake (PMTDI) for AFB_1_ because it is a genotoxic carcinogen, and the recommendation is to minimize exposure as much as possible^35^^)^. For a global perspective, over 100 nations have set regulatory limits on allowable AF in food and feed, and most countries regulate sum totals of B_1_, B_2_, G_1_, and G_2_. There are no specific regulations for AF in pulses worldwide; however, regulations are set broadly for human food consumption. The ML for Codex Alimentarius are 10 ppb (µg/kg) for total AF in ready to eat peanuts and tree nuts, 5 ppb (µg/kg) for AFB_1_ in cereals and cereal-based foods, and 0.5 ppb (µg/kg) of AFM_1_ in milk^36^^)^.

#### 2.1.2 Ochratoxin A (OTA)

Ochratoxin is a mycotoxin produced by certain species of *Aspergillus ochraceous* and *Penicillium verrucosum*^37^^)^. Ochratoxin A (OTA) is the most prominent member of this toxin family (including ochratoxin B and C), and dietary exposure can cause a myriad of health issues including poultry ochratoxicosis, porcine nephropathy, urinary tract tumors, and endemic nephropathies in humans^38^^,^^39^^,^^40^^)^. OTA is highly abundant in food and feed, and it is frequently detected in all types of cereals and cereal byproducts, coffee, cacao, grapes, raisins, wine, soybeans, spices, nuts, pulses, and beer^41^^)^.

Guidance values for OTA have been issued by the EU, including Codex Alimentarius proposed limits, for cereals and cereal products at 5 ppb (µg/kg), processed cereals at 3 ppb (µg/kg) for human consumption, wine and grape juice at 2 ppb (µg/kg), roasted coffee beans at 5 ppb (µg/kg), and dried vine fruits at 8-10 ppb (µg/kg)^36^^,^^37^^,^^42^^,^^43^^)^. The US has no set levels for OTA in food or feed products, but OTA is monitored through the FDA’s compliance program^44^^)^. China has established limits for OTA at 5 ppb (µg/kg) for beans, grains, grain-based products, and roasted and ground coffee, and 2 ppb (µg/kg) for wine^33^^)^. Japan has conducted a risk assessment and deemed OTA as having no apparent adverse effect on humans through food, with overall low health risk in relation to the levels of exposure^45^^)^.

#### 2.1.3 Cyclopiazonic acid (CPA)

CPA is a mycotoxin produced by certain species of *Aspergillus* and *Penicillium*. It has been identified in several food sources including cereals, legumes, milk, meat, and cheese^46^^)^. CPA is a tremorgenic mycotoxin causing symptoms of weight loss, fever, diarrhea, dehydration, ataxia, immobility, and muscle spasms. It affects the gastrointestinal tract, liver, spleen, and muscle tissues^46^^)^. CPA mycotoxicosis is benign in nature compared to other mycotoxins such as AF, and it is not considered a potent acute toxin^46^^)^. The FDA has not established regulatory guidelines for CPA.

#### 2.1.4 Aflatrem

Aflatrem is a major tremorgenic mycotoxin produced by *A. flavus,* which is mainly concentrated in the sclerotia^47^^)^. It is a potent neurotoxin, known to cause tremors and neurological disorders, including mental confusion, seizures, and hyperexcitability, in rats and cattle^48^^)^. This mycotoxin poses significant agricultural and health problems for both livestock and humans. The production of aflatrem is regulated by the *veA* gene, which is also involved in the production of other mycotoxins such as AF and CPA^49^^)^. There are no regulatory limits globally for aflatrem, or other indole diterpenes, such as aflavinine.

#### 2.1.5 Ustiloxins

Ustiloxins are a family of cyclic tetrapeptide mycotoxins first derived from the phytopathogenic fungus *Ustilaginoidea virens*. Since the discovery ustiloxins have also been found to be produced by *Aspergillus* and *Diaporthe* spp^50^^)^. Ustiloxins are similar in structure to phomopsins (see 2.8.1) and exhibit potent antimitotic activity by inhibiting microtubule assembly^51^^,^^52^^)^. Ustiloxins, particularly ustiloxin A and B, accumulate in rice false smut balls and can impair growth and seed germination, posing risks to crop productivity and food safety^52^^,^^53^^)^. At present, there are no food safety authorities that have established maximum limits, or monitoring requirements for ustiloxins in food or feed.

### 2.2 *Fusarium* spp.

**Fig. 3.**
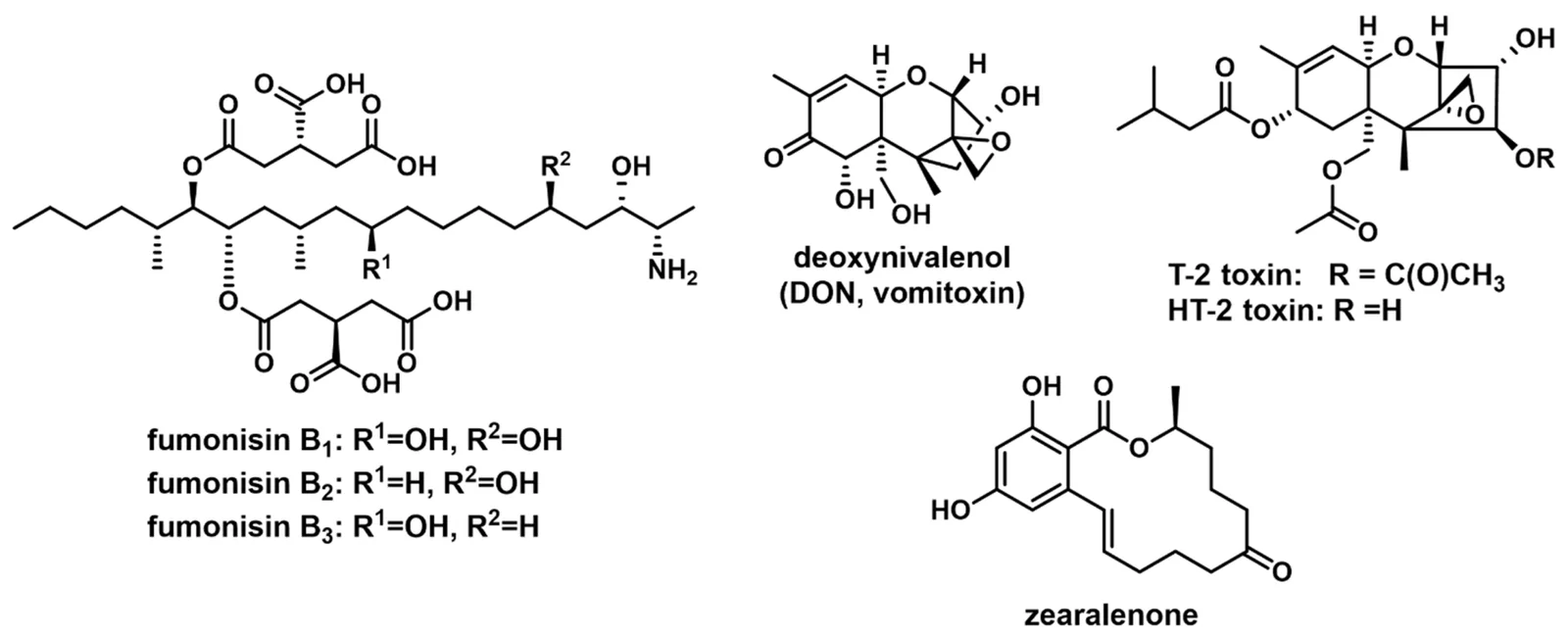
**Molecular structures for *Fusarium* spp. toxins.** There are seven major toxins, fumonisins B_1_, B_2_, and B_3_, deoxynivalenol also known as vomitoxin, T-2 toxin, HT-2 toxin, and zearalenone, produced by *Fusarium* spp.

*Fusarium* spp. are a group of filamentous fungi that produce a wide variety of diseases in agricultural production, including root rots, wilts, crown rots, and head blight^54^^,^^55^^)^. These fungal species produce three primary types of mycotoxins: trichothecenes (type A: T-2, HT-2, and type B: deoxynivalenol (DON)), fumonisins (FUMs), and zearalenone (ZEN) (**Fig. 3**)^56^^)^. *Fusarium verticillioides* and *Fusarium proliferatum* primarily produce FUM, whereas *Fusarium graminearum* and *Fusarium culmorum* produce DON and ZEN. Compared to other commodities, including cereal grains like corn, pulses accumulate low amounts of FUMs, DON, and ZEN^16^^)^.

#### 2.2.1 Fumonisins (FUMs)

FUM is a mycotoxin commonly found in corn and other cereals such as wheat, barley, and oats, making it one of the most widespread mycotoxins worldwide^57^^)^. There are three major types of FUMs—FB_1_, FB_2_, and FB_3_—with FB_1_ being the most prevalent and toxic. The IARC has classified FB_1_ as a Group 2B carcinogen, indicating it is probably carcinogenic to humans^58^^)^. Exposure to FB_1_ has been linked to several severe health issues, including leukoencephalomalacia (LEM) in horses, pulmonary edema syndrome (PES) in pigs, and an elevated risk of esophageal cancer in humans^57^^)^. Additionally, FUM can disrupt the metabolism of sphingolipids, which are essential components of cell membranes, leading to various cellular dysfunctions^59^^)^.

Despite FUM being classified as neurotoxic, hepatotoxic, and nephrotoxic, the US has not set action levels. However, guidance levels for FUM contamination in corn have been set. The recommended guidance levels are 2 ppm (mg/kg) for total FUMs (FB_1_+FB_2_+FB_3_) for human consumption in degermed dry milled corn products, 3 ppm (mg/kg) for cleaned corn for popcorn, 4 ppm (mg/kg) for whole or partially degermed dry milled corn products, dry milled corn bran and cleaned corn for masa production. The levels vary for animal feed depending on the species and stage of production: 30 ppm (mg/kg) for breeding livestock, 20 ppm (mg/kg) for swine and catfish, and 5 ppm (mg/kg) for horses and rabbits^60^^)^. The EU has set ML for FUM (sum of B_1_ and B_2_) in cereal and cereal-based by-products at 1 ppm (mg/kg) in maize and maize-based foods for the final consumer, 4 ppm (mg/kg) in unprocessed maize (except for wet-milling use), and 200 ppb (µg/kg) for baby food containing maize and processed maize^32^^,^^43^^)^. China regulates FUM in maize at 4 ppm (mg/kg), 2 ppm (mg/kg) in maize flour and starch and 1 ppm (mg/kg) for grain products containing maize^33^^,^^34^^)^. The Food Safety Commission of Japan (FSCJ) has stated that FUM exposure through food for humans is unlikely^61^^,^^62^^)^. Codex Alimentarius, informed by JEFCA, established ML for FUM in raw maize and processed maize ranging from 2-4 ppm (mg/kg), depending on the product type. Australia and New Zealand follow Codex recommendations^36^^)^.

Guidelines for FUM levels in pulses are less common compared to the regulations for corn, wheat, barley, and oats. The FDA and the EU have set guidance levels for overall animal feed, but no specific regulations for pulses have been evaluated. While there are no stringent regulations for FUM globally for pulses, it is critical to monitor and manage FUM to ensure overall food and feed safety.

#### 2.2.2 Deoxynivalenol (DON)

DON, also known as vomitoxin due to its strong emetic effects, is a naturally occurring mycotoxin in corn, wheat, barley, and their coproducts^63^^,^^64^^)^. All animal species evaluated to date—including rodents, pigs, dogs, cats, poultry, ruminants, and humans—are susceptible to DON. This mycotoxin disrupts normal cell function by inhibiting protein synthesis, binding ribosomes, and activating cellular kinases involved in signal transduction related to proliferation, differentiation, and cellular apoptosis^65^^)^. Epidemiological studies suggest that chronic exposure to DON may result in reduced growth, impaired immune function, and reproductive issues^65^^)^.

Regulatory limits for DON vary globally. The FDA and Canadian guidelines have set advisory levels for DON in finished wheat products (flour, bran, and germ) for human consumption at 1 ppm (mg/kg). For animal feeds, the levels vary: 10 ppm (mg/kg) for poultry in grain and grain byproducts not exceeded at 50% of the diet, 5 ppm (mg/kg) for swine not exceeded at 20% of the diet, 10 ppm (mg/kg) for feedlot cattle, and 5 ppm (mg/kg) for other animals not exceeded at 40% of the diet^66^^)^. EU regulations have established ML for DON at 1.5 ppm (mg/kg) for unprocessed durum wheat and maize grains, 1 ppm (mg/kg) for unprocessed cereals, 0.75 ppm (mg/kg) for final consumer cereal and milled maize products, and maize for popcorn, and 1.75 ppm (mg/kg) for unprocessed oat grains with an inedible husk^32^^,^^43^^,^^67^^)^. China regulates DON in grains and grain-based foods at 2 ppm (mg/kg) for grains, 1 ppm (mg/kg) for grain flour and 0.2 ppm (mg/kg) in cereal-based infant foods^33^^)^. For food in Japan, the FSCJ has set 1 μg/kg/day for the TDI of DON^34^^,^^68^^,^^69^^)^. Similar to FUM, there are no specific regulations for DON in pulses set by major regulatory bodies globally^36^^,^^70^^)^. The Codex Alimentarius Commission permits up to 0.2 ppm (mg/kg) of DON in cereal-based foods for infants and young children; 1 ppm (mg/kg) DON in flour, meal, semolina, or flakes made from wheat, maize, or barley; and 2 ppm (mg/kg) DON in wheat, maize, and barley destined for further processing^36^^)^.

#### 2.2.3 Zearalenone (ZEN)

ZEN is produced in several species of *Fusarium*, particularly *F. graminearum* and *F. culmorum*^71^^)^. It is a non-steroidal estrogenic mycotoxin, and it is known for causing reproductive issues in livestock such as infertility, abortion, and other breeding problems^72^^)^. The animal species most sensitive to ZEN are swine and ruminants. In humans, long-term exposure to ZEN can pose significant health risks, such as prostate, ovarian, cervical, or breast cancers^73^^)^. ZEN often coexists with DON and are frequent contaminants of corn, wheat, oats, and barley^74^^)^.

The EU has provided guidance levels for ZEN in compound feed at 0.1 ppm (mg/kg) for piglets and gilts, 0.25 ppm (mg/kg) for sows and fattening pigs, 0.50 ppm (mg/kg) for calves, kids, lambs, sheep, goats, and dairy cattle^75^^)^. The EU has ML of ZEN at 100 ppb (µg/kg) for unprocessed cereal grains, excluding maize grains, which are set at 350 ppb (μg/kg). Cereals, bran, germ, cereal flour and semolina for the final consumer have a ML of 75 ppb (μg/kg)^32^^,^^43^^)^. The FDA does not have specific guidelines established for ZEN in grains or food/feed products^76^^,^^77^^)^. The Codex Alimentarius has developed guidance based on JEFCA at PMTDI: 0.5 µg/kg bw/d for total intake of zearalenone and its metabolites (including alpha-zearalanol) in foodstuffs^78^^)^. China regulates cereals at 60 ppb (μg/kg) for grain and grain byproducts^33^^)^. Japan limits ZEN to1 ppm (mg/kg) for feed and 0.5 ppm (mg/kg) for formula feed^34^^)^.

#### 2.2.4 T-2 and HT-2

T-2 and HT-2 toxins are type A trichothecenes produced by many fungi, but *Fusarium spp.* are most important due to their ubiquity^79^^)^. These fungi commonly infect small grain cereals such as oats, barley, wheat, and maize, especially in cold temperate climates. Because of T-2 and HT-2’s similar toxicological profiles, they are usually assessed together as a combined exposure group. T-2 and HT-2 inhibit protein synthesis, leading to cytotoxic, immunosuppressive, and dermatotoxic effects in animals. A historic outbreak of alimentary toxic aleukia (ATA) in humans from 1932 to 1947 in the former USSR, linked to consumption of overwintered grains, highlighted the severe toxicity of type A trichothecenes, although such events are now rare due to modern grain handling practices^79^^)^.

The EFSA established a group TDI of 0.02 µg/kg bw for combined T-2 and HT-2 exposure in 2017^80^^,^^81^^)^. Additionally, ML have been set in the EU for sum of T-2 and HT-2 toxins, ranging from 1.25 ppm (mg/kg) for oat grains with inedible husks to 10 ppb (μg/kg) for cereal-based foods used for infants and children, and 0.05 ppm (mg/kg) for compound feed for cats^32^^,^^43^^,^^75^^,^^82^^,^^83^^)^.

### 2.3 *Penicillium* spp.

**Fig. 4.**
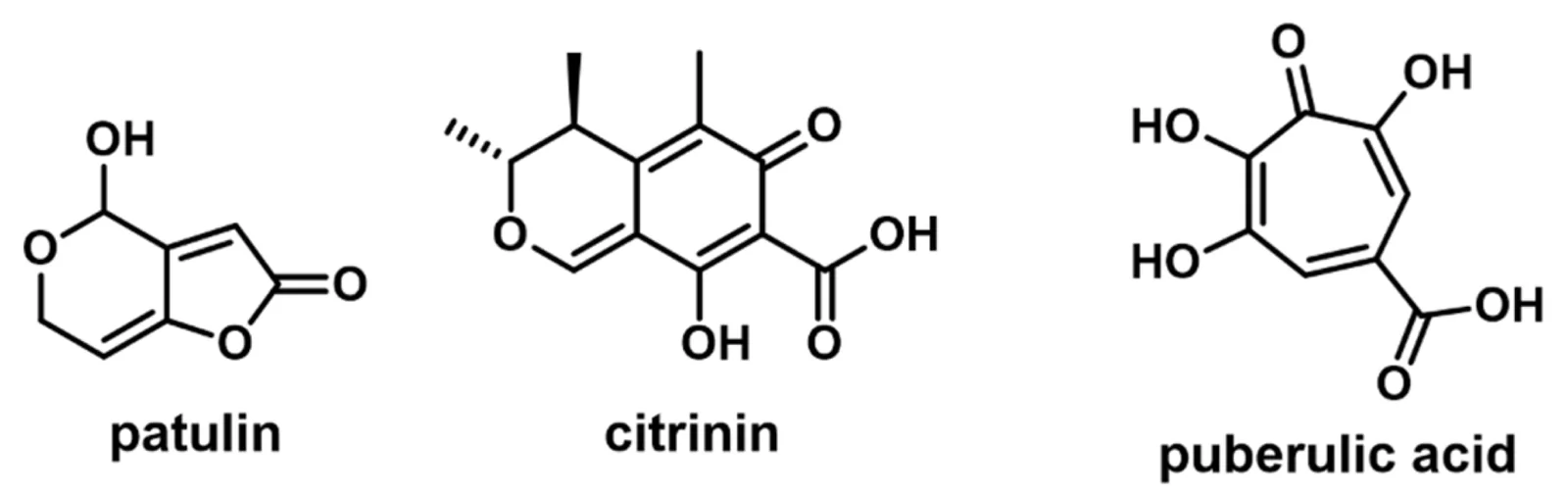
**Molecular structures for *Penicillium* spp. toxins.** There are three major toxins, patulin, citrinin, and puberulic acid, produced by *Penicillium* spp.

*Penicillium* spp. are a diverse and cosmopolite fungi, and there are 350 species recognized within this genus. It is one of the most common fungi occurring worldwide, and the species are frequently associated with spoilage of foods and feeds as well as isolated from soil^84^^)^. There are many mycotoxins produced across *Penicillium* spp. that demonstrate some level of toxicity. Of these mycotoxins there is OTA and CPA, which were discussed previously (see 2.1.2 and 2.1.3), in addition to patulin, citrinin, and puberulic acid (PA) (**Fig. 4**)^85^^)^.

#### 2.3.1 Patulin

Patulin is primarily produced by *Penicillium expansum*, a fruit pathogen, which commonly causes apple rot. It can contaminate other fruits, vegetables, and their derived coproducts. It is known for its nephrotoxic, neurotoxic, hepatotoxic, and immunosuppressive effects in both humans and livestock^86^^)^.

The ML, based on Codex Alimentarius and EU, for patulin are 50 ppb (μg/kg) in apple juices and apple-based drinks, 25 ppb (μg/kg) in solid apple products, and 10 ppb (μg/kg) in infant and baby food^32^^,^^36^^)^. The FDA has set action levels for patulin in apple juice and apple juice concentrate at 50 ppb (μg/kg)^87^^)^. China has less strict limits than Codex and the EU, with 50 ppb (μg/kg) being the limit for fruit, fruit products, beverages and liquor^33^^)^. The WHO has set a PMTDI for patulin at 0.4 ppb (μg/kg) bw/d^88^^)^. To date patulin has been rarely assessed or detected in pulse crops.

#### 2.3.2 Citrinin

Citrinin is mainly produced by *Penicillium citrinum*, but both *P. expansum* and *Penicillium verrucosum* may also produce the toxin^89^^)^. It is structurally similar to OTA and exhibits nephrotoxic properties including kidney dysfunction^90^^)^. Citrinin has been identified in various food sources including fermented sausages, wheat, corn, rice, and meals colored with *Monascus* pigments^90^^,^^91^^)^. It can contaminate pulses under certain storage conditions, such as improper storage or high humidity; however, it is more common in cereals^92^^,^^93^^)^.

The regulatory guidelines for citrinin vary globally; the EU has established ML for citrinin for food supplements, including red yeast rice (RYR), at 100 ppb (µg/kg)^32^^,^^94^^)^. The FDA has not established regulatory limits for citrinin in food products and warns consumers against RYR products due to some products containing an unauthorized drug^95^^,^^96^^)^. China limits citrinin in rice and red yeast rice products at 50 ppb (μg/kg)^91^^)^.

#### 2.3.3 Puberulic acid (PA)

PA is a highly oxygenated polyketide defined by a tropolone ring isolated from *Penicillum puberulum* and related *Penicillium* species. PA and its analogs exhibit potent antimicrobial properties, notably antimalarial activity against *Plasmodium falciparum*^97^^)^. Despite the promising bioactivity, it also shows significant cytotoxicity, as described in a major food poisoning report from Japan in March 2024. Contaminated red yeast rice (RYR), or beni-koji in Japan, caused kidney damage from PA-producing *Penicillium adametzioides*^98^^,^^99^^,^^100^^,^^101^^)^. Although not typically classified as a major foodborne mycotoxin, this recent outbreak underscores the need for further investigation into fungal secondary metabolites. To date, there are no regulatory limits established for PA.

### 2.4 *Alternaria* spp.

**Fig. 5.**
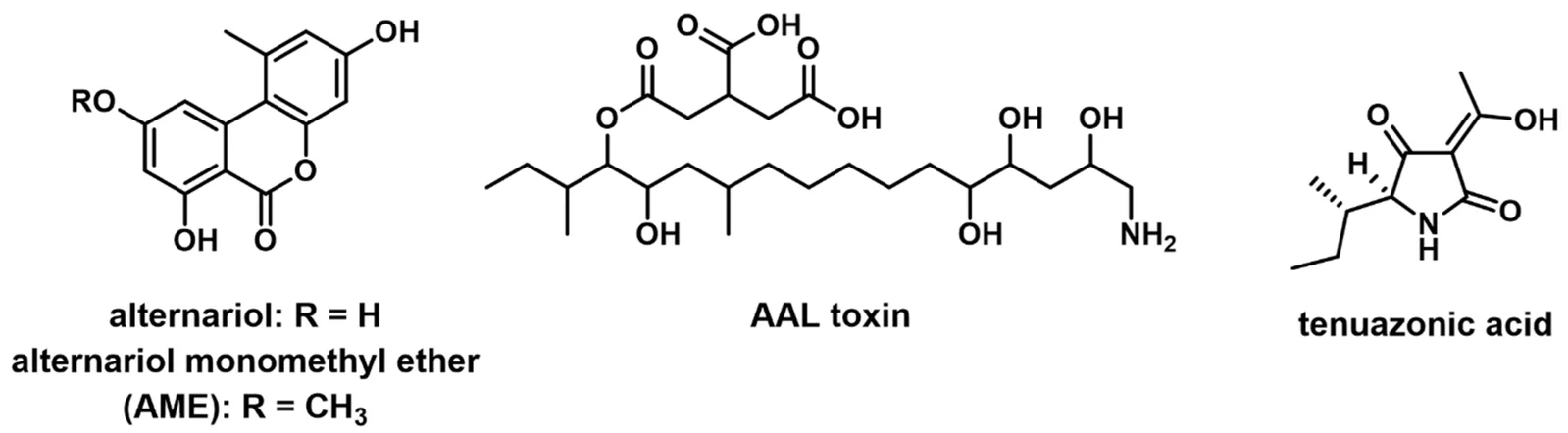
**Molecular structures for *Alternaria* spp. toxins.** There are four major toxins, alternariol, alternariol monomethyl ether (AME), AAL toxin and tenuazonic acid, produced by *Alternaria* spp.

The genus *Alternaria* includes more than 250 species, and they can produce a wide variety of toxic metabolites. It was found by the EFSA that “legumes, nuts and oil seeds” food group had the highest concentrations of *Alternaria* toxins, particularly sunflower seeds. But there is limited information on how these toxins ultimately impact food and feed supply chains^102^^)^. For our review we will cover a few common mycotoxins produced by *Alternaria* spp.: alternariol (AOH), alternariol monomethyl ether (AME), tenuazonic acid (TeA), and AAL toxins (**Fig. 5**)^102^^,^^103^^)^.

#### 2.4.1 Alternariol (AOH)

AOH is produced by *Alternaria* fungi, and it naturally occurs in foodstuffs including fruit and grain products^104^^)^. AOH toxicity and potential mechanisms have been studied *in vitro*; however, there is very limited *in vivo* analyses available. AOH has been noted to form reactive oxygen species (ROS), interacting with DNA topoisomerase, thus triggering single and double-strand DNA breaks. This results in various DNA damage response pathways^104^^)^. Acute toxicity of AOH exists, but it is low compared to other mycotoxins. No experimental studies have been performed with animals to clarify possible esophageal cancer risk^102^^)^.

The EU has set indicative levels for AOH in various food products in 2022, including cereal-based foods for infants and young children at 2 ppb (μg/kg), processed tomato products and sunflower oil at 10 ppb (μg/kg), and seeds (sesame, sunflower) at 30 ppb (μg/kg)^83^^)^. However, these indicative levels are not food safety levels.

#### 2.4.2 Alternariol monomethyl ether (AME)

AME has been reported as cytotoxic and mutagenic and may be more cytotoxic than AOH in certain instances. Further studies need to be done to assess *in vitro* analysis of AME and possible synergistic or antagonistic effects *in vitro* in conjunction with AOH and TeA^105^^)^.

The EU has set indicative levels of AME, which are the same as AOH levels mentioned above, except for processed tomato products, which is set at 5 ppb (μg/kg)^83^^)^. It is important to note these are indicative levels and have only been set where there is significant occurrence data.

#### 2.4.3 Tenuazonic acid (TeA)

TeA has been reported as cytotoxic, however it has been reported to be 3-5 times less cytotoxic than AOH and AME. TeA has been found to inhibit protein release from ribosomes and decreased cell viability. Overall, further research needs to be done to examine the *in vitro* effects of TeA^105^^)^.

The EU has also set indicative levels for TeA, which are much less strict compared to the previous *Alternaria* mycotoxins discussed. Levels are set at 100 ppb (μg/kg) for sesame seeds, sunflower oil, and tree nuts. Cereal-based food for infants and young children, and processed tomato product levels are set to 500 ppb (μg/kg). Dried fig and sunflowers seed levels are set to 1000 ppb (μg/kg) and paprika powder is set to 10,000 ppb (μg/kg)^83^^)^. Again, these indicative levels are not strict regulations.

#### 2.4.4 AAL toxins

AAL toxins are a group of host-specific toxins produced by *Alternaria alternata* f. sp. *lycopersici*, which is a common cause of tomato stem canker disease^106^^)^. AAL toxins are structured similarly to FUM and are known to disrupt cellular homeostasis in both plant and animal tissues^107^^)^. Currently, there are no specific regulatory limits for AAL toxins set by major regulatory bodies including the FDA or EU.

### 2.5 *Rhizopus microsporus*

**Fig. 6.**
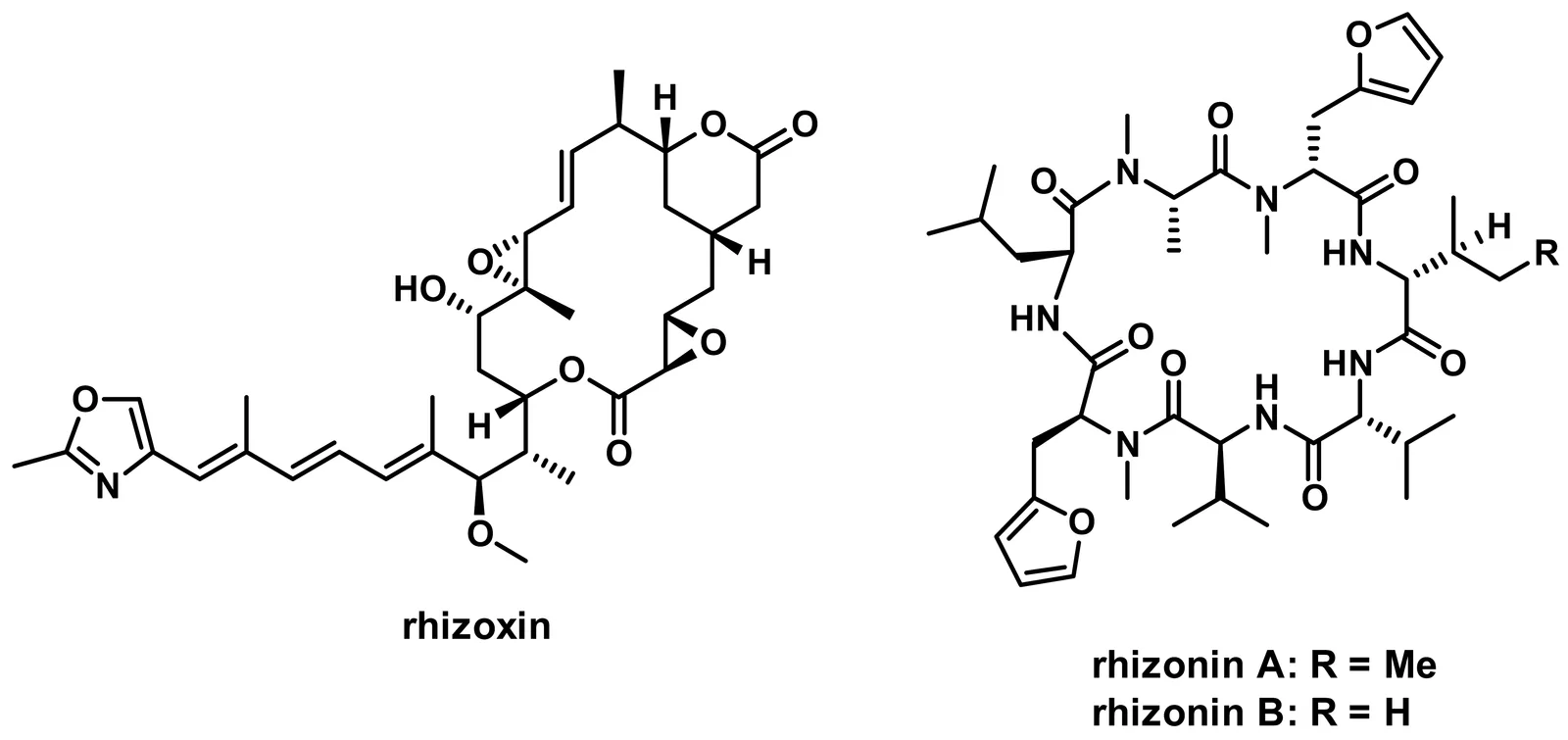
**Molecular structures for *Rhizopus microsporus* toxins.** There are three major toxins, rhizoxin, and rhizonin A and B, produced by *Rhizopus microsporus*.

*Rhizopus microsporus* is a fungal plant pathogen that affects corn, rice, and sunflowers. It causes rice seedling blight and *Rhizopus* head rot in sunflowers^108^^)^. It produces several mycotoxins, including rhizoxin and rhizonins (**Fig. 6**).

#### 2.5.1 Rhizoxin

Rhizoxin is a hepatotoxic cyclopeptide isolated from *R. microsporus*. It is the lead causative agent of rice seedling blight^109^^)^. It is not a fungal metabolite unlike the other mycotoxins; however, it is bacterial symbiont living inside the fungi. Rhizoxin disrupts microtubule formation in eukaryotic cells; thus, inhibiting cellular division and potentially has anti-tumor properties^110^^)^.

#### 2.5.2 Rhizonin

Rhizonins A and B are cyclopeptide toxins produced by a fungal endobacteria, first discovered in contaminated Mozambican peanuts^110^^)^. Further research is needed to understand the relationship between fungi like *R. microsporus*, and endofungal bacteria, which can potentially have health-threatening properties to humans and livestock when producing toxins^109^^)^.

### 2.6 *Macrophomina phaseolina*

**Fig. 7.**
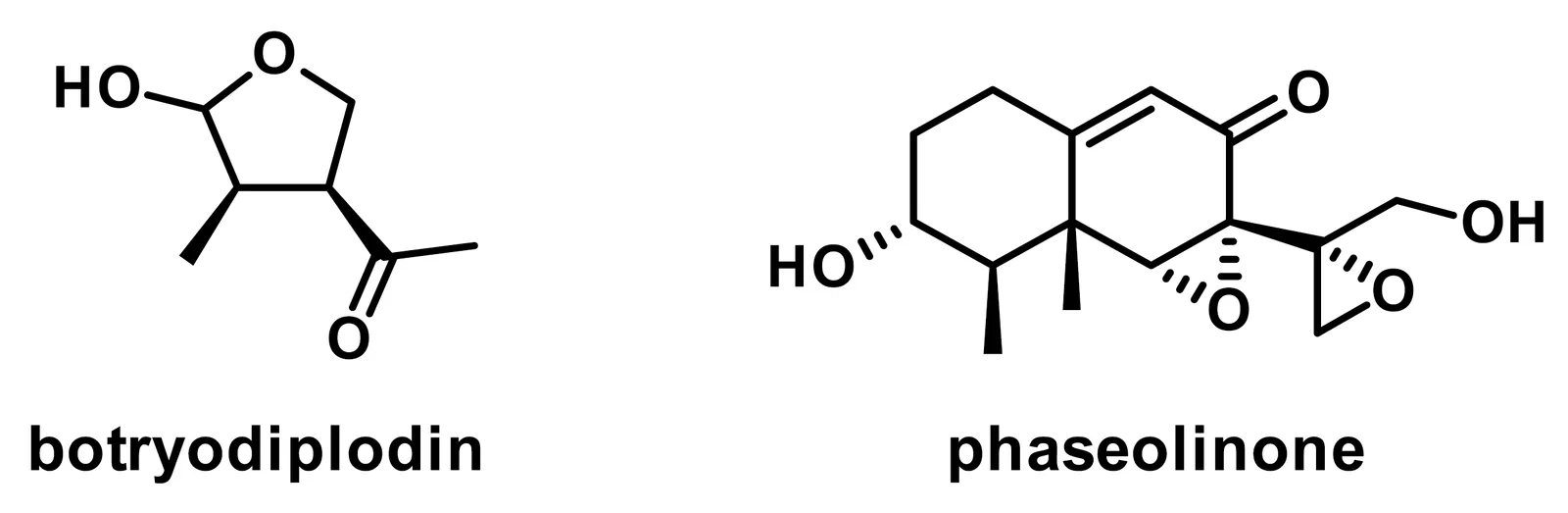
**Molecular structures for *Macrophomina phaseolina* toxins.** There are two major toxins, botryodiplodin and phaseolinone, produced by *Macrophomina phaseolina*.

*Macrophomina phaseolina* is a soil-borne fungus found globally, and it causes a myriad of diseases including stem, root, and charcoal rot, and seedling blight in soybeans, sorghum, and groundnuts^111^^)^. Generally, this fungus is present under high temperatures (30-35 °C) and low soil moisture (below 60%), leading to significant yield losses^112^^)^. *M. phaseolina* produces toxins botryodiplodin and phaseolinone (**Fig. 7**). These toxins have not been assessed by the FDA or EU for food safety limits.

#### 2.6.1 Botryodiplodin

Botryodiplodin, a mycotoxin produced from *M. phaseolina,* causes charcoal rot disease in various crops, including soybeans^113^^)^. It is a ribose-analog toxin, meaning it structurally resembles ribose, a crucial component of RNA and ATP^114^^)^. This mycotoxin exhibits anticancer, antibacterial, antifungal, phytotoxic, mitogenic, and antifertility components^114^^)^. Different plant cultivars show varying susceptibility to botryodiplodin, with some being more resistant than others.

#### 2.6.2 Phaseolinone

*M. phaseolina* (Tassi) Goid., the causal agent of charcoal rot disease of soybean, can cause disease in more than 500 other commercially important plants^115^^)^. Phaseolinone, a fungal secondary metabolite, is an understudied mycotoxin that inhibits RNA polymerases^115^^,^^116^^)^.

### 2.7 *Cercospora kikuchii*

**Fig. 8.**
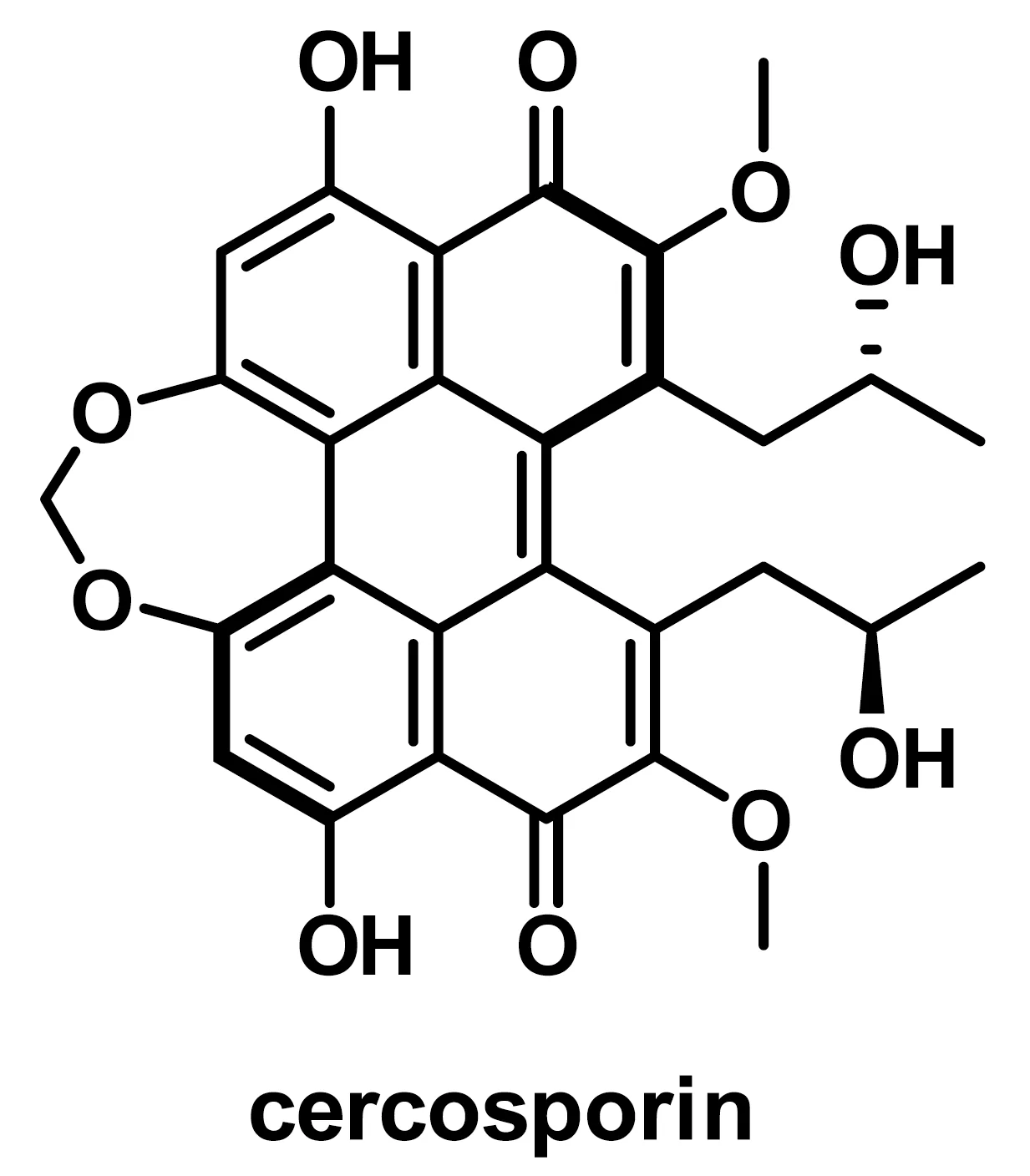
**Molecular structure for *Cercospora kikuchii* toxin.** There is one major toxin, cercosporin, produced by *Cercospora kikuchii*.

*Cercospora kikuchii* is a fungal pathogen responsible for Cercospora leaf blight and purple seed stain in soybeans^117^^)^. Additionally, cercosporin is a photoactivated toxin produced from the genus *Cercospora* (**Fig. 8**). Cercosporin has not been assessed by the FDA or EU for food safety limits.

#### 2.7.1 Cercosporin

Cercosporin is the photoactivated toxin produced by several fungal plant pathogens, including species of *Cercospora*, *Alternaria*, *Cladosporium*, and *Elsinoe*^118^^)^. Cercosporin is a red pigment and a type of perylenequinone. This mycotoxin is inactive in the dark, but it becomes toxic when exposed to light. Upon exposure, cercosporin generates ROS, including singlet oxygen and superoxide causing oxidative damage to cellular components^118^^)^.

### 2.8 *Diaporthe toxica*

**Fig. 9.**
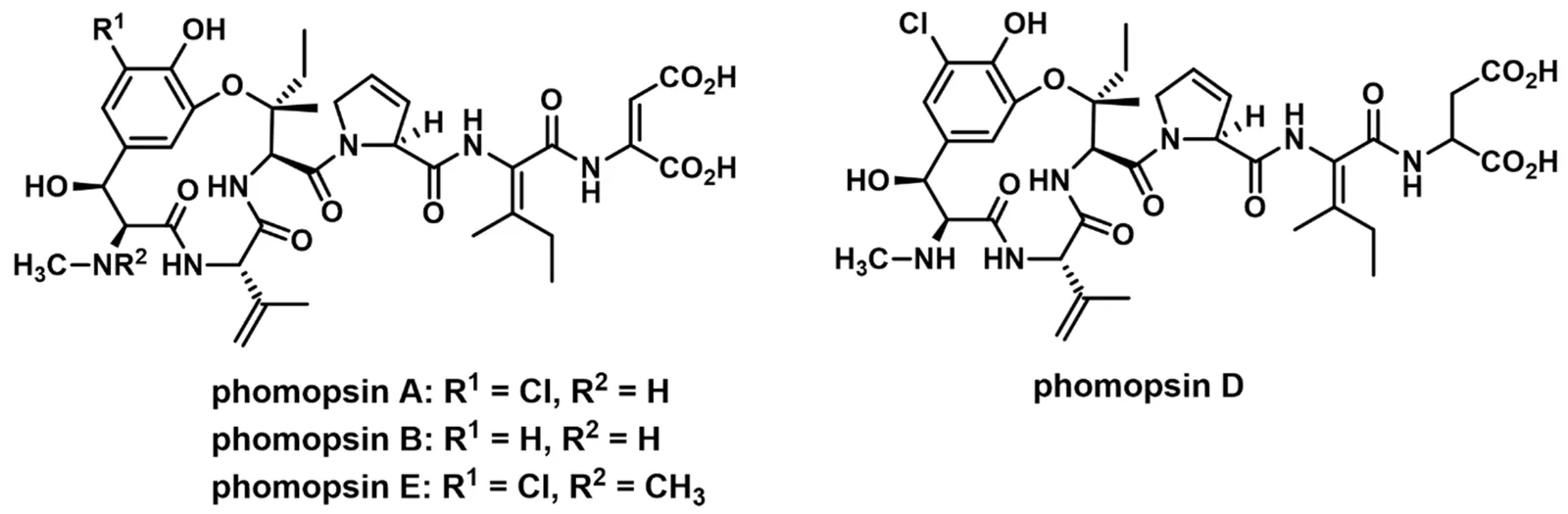
**Molecular structures for *Diaporthe toxica* toxins.** The major toxins produced by *Diaporthe toxica*, previously named *Phomopsis leptostromiformis,* are phomopsins. Pictured are structures for phomopsin A, B, D, E. To our knowledge, the structure of phomopsin C has not been reported^180^^)^.

*Diaporthe toxica,* formerly known as *Phomopsis leptostromiformis*, is a saprotrophic fungus that synthesizes mycotoxins under high humidity^119^^)^. This fungus is an emerging threat, specifically for lupine, a pulse crop with similar allergenicity to peanuts.^120^^)^ It causes stem and pod blight in *Lupinus* spp., and it produces a group of mycotoxins known as phomopsins (**Fig. 9**) and ustiloxins (see 2.1.5)^16^^,^^119^^)^.

#### 2.8.1 Phomopsins (PHOs)

PHOs produced by *Diaporthe toxica,* particularly phomopsin A (PHO-A), cause lupinosis^16^^,^^121^^)^. Several other phomopsins including PHO-B, PHO-C, PHO-D, and PHO-E have also been identified, but there is limited research on their individual toxicity^121^^)^. PHO-A is hepatotoxic across animal species, hepatocarcinogenic in rats, and nephrotoxicity is more common in pigs and horses^122^^)^. PHOs cause severe liver damage, lethargy, loss of appetite and death, especially when fed as dry forage to livestock^121^^)^. These symptoms are classified as lupinosis and can be avoided by growing *Diaporthe* resistant cultivars^121^^)^.

The European Food Safety Authority’s (EFSA) report concluded that more information is needed about the extent of lupine consumption in humans and livestock for accurate food safety levels to be established in the EU. The same could be argued for the US, as lupine is slowly becoming more popular. It is currently noted that in Australia, a top producer of lupine, it is unlikely that levels of PHO exceed 5 ppb (μg/kg). Because of these findings, the current ML of 5 ppb (μg/kg) for PHOs in food products remains in Australia, New Zealand and the FAO^122^^)^.

### 2.9 Other Documented Nontoxigenic Fungi Infecting Pulses

Nontoxigenic fungi can also infect pulses, leading to various agricultural and quality issues. Unlike mycotoxigenic fungi, which produce harmful mycotoxins, nontoxigenic fungi do not pose direct health risks. However, their presence can still negatively impact crop yield and quality. These fungi include *Mucor* spp., *Phytophthora infestans*, *Curvularia spicifera* formerly known as *Drechslera tetramera*, and *Rhizopus stolonifer*. *Mucor* spp. consists of 40 species of molds in the family *Mucoraceae*. These fungi are commonly found in soil, plants, dairy products, and decaying fruits and vegetables^123^^)^. *Mucor* spp. can also be found in cereals and grains, but they are less serious filamentous fungal pathogens compared to other mycotoxigenic species^123^^)^. *P. infestans* is a plant pathogen that is the causal agent of potato late blight and tomato blight^124^^)^. It is an oomycete, and the pathogen produces sporangia and zoospores^125^^)^. *C. spicifera* is a filamentous fungi belonging to the Ascomycota phylum, and it can infect a wide variety of plants including sunflowers and cereal grains^126^^)^. *R. stolonifer*, commonly known as black bread mold, is a widespread fungus belonging to the phylum Mucormycota^127^^,^^128^^)^. It is considered a major destructive postharvest disease to stored fruits and vegetables. Despite their nontoxigenic nature, managing these fungal infections is crucial to maintaining the quality and safety of pulses throughout the supply chain.

## 3. Changes in Pulse Nutrient Levels as Affected by Pathogenic Fungi

Pulses are grown globally and are a commodity most known for their high protein content (>20g/100g dry matter)^129^^)^. In 2013, the United Nations declared 2016, the “International Year of Pulses”, and 2 years later in 2018, they declared that February 10^th^ would be World Pulses Day, with the first recognized day being February 10, 2019^130^^)^. In a 10-year study comparing pulse consumers to non-consumers in the US, Mitchell and company (2021) found that pulse consumption resulted in a more nutrient dense diet.^131^^)^ Across the US, production of pulses is at a high, however, they are still produced much less than other plant-based proteins (namely peanuts and soybeans) (**Table 1**).

**Table 1.** United States production of pulses in 2024 (CWT), compared to peanuts (lb) and soybeans (bu)^135^^)^.

**State**	**Dry bean**	**Chickpea**	**Dry pea**	**Lentil**	**Peanuts (lb)**	**Soybeans (bu)**
Colorado	980,000 (4.98 x 10^7^)^a^	–	–	–	–	–
Idaho	1,056,000 (5.36 x 10^7^)	1,193,000 (6.06 x 10^7^)	210,000 (1.07 x 10^7^)	–	–	–
Michigan	6,448,000 (3.28 x 10^8^)	–	–	–	–	111,180,000 (3.03 x 10^9^)
Minnesota	5,440,000 (2.76 x 10^8^)	–	–	–	–	337,180,000 (9.18 x 10^9^)
Montana	–^b^	2,205,000 (1.12 x 10^8^)	10,679,000 (5.43 x 10^8^)	7,038,000 (3.58 x 10^8^)	–	–
Nebraska	2,541,000 (1.29 x 10^8^)	–	470,000 (2.39 x 10^7^)	–	–	309,750,000 (8.43 x 10^9^)
North Dakota	11,715,000 (5.95 x 10^8^)	786,000 (3.99 x 10^7^)	7,410,000 (3.76 x 10^8^)	2,000,000 (1.02 x 10^8^)	–	250,800,000 (6.83 x 10^9^)
Washington	1,268,000 (6.44 x 10^7^)	1,948,000 (9.9 x 10^8^)	509,000 (2.59 x 10^7^)	500,000 (2.54 x 10^7^)	–	–
**US Total**	**29,448,000(1.5 x 10^9^)**	**6,132,000(3.12 x 10^8^)**	**19,278,000(9.79 x 10^8^)**	**9,538,000(4.85 x 10^8^)**	**6,512,300,000 (2.95 x 10^9^)**	**4,461,310,000(1.21 x 10^11^)**

^a^Values in parentheses were converted to kg based on original reported values in hundredweight (CWT) for dry bean, chickpea, dry pea and lentil; pounds (lb) for peanuts and bushels (bu) for soybeans. Conversions are as follows: 1 CWT = 50.8023 kg; 1 soybean bu = 27.22 kg; 1 lb = 0.453592 kg.

^b^–, no data available/not reported to be grown in this area.

**Fig. 10.**
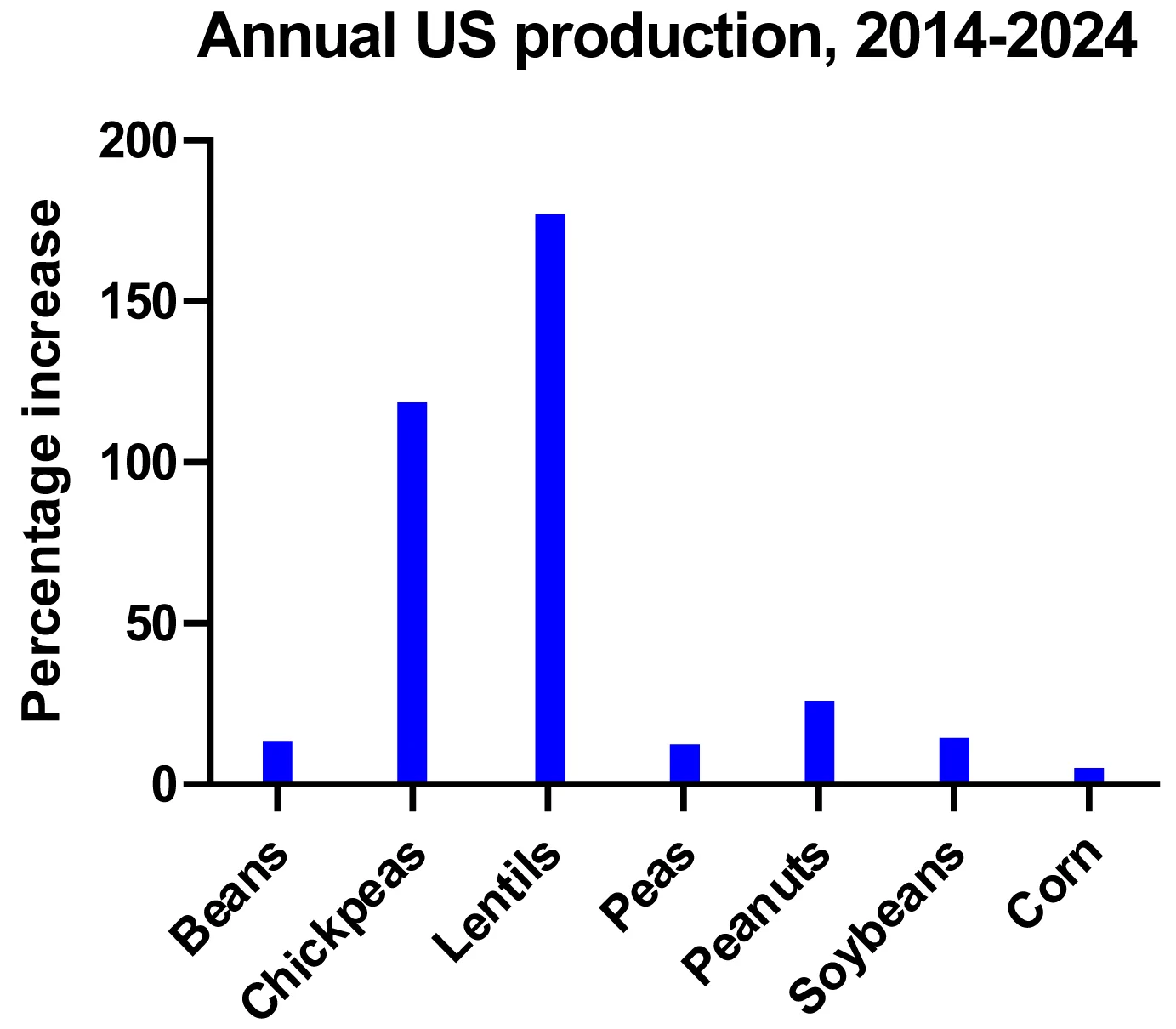
**Annual production of pulses in the United States from 2014 compared to 2024.** Pulses, including beans, chickpeas, lentils and peas are commodities in the United States and compared to ten years ago (2014), there is an increase in production of all pulse crops, most notably the over 100% increase in chickpea and lentil production. Also included are peanuts, and soybeans, well-established plant proteins, and corn as a popular grain in the US. This may demonstrate that these crops are becoming more important as the search for sustainable plant-based proteins increases^135^^)^.

Over the past decade in the US (2014-2024), pulse crop production has increased (Fig. 10). This growth could be attributed to several factors, increased nutrient density, declarations by the UN, or that there is a need to grow more food for more people as the global population increases^2^^,^^130^^,^^131^^)^. Recently, Messina and colleagues discussed the nutrition of pulses globally, as well as their main uses in foods, but did not address changes in nutritional quality, those causes or related outcomes^132^^)^. Despite these factors, further research on pulses is necessary, particularly in areas such as nutritional composition, fungal infection and mycotoxin contamination. We aim to address these gaps in the following sections.

By examining the whole nutritional profile of pulses, especially when infected with fungi, we can get a better understanding of how the seeds change in response to fungal threats. For instance, a review by Sammoud et al. discusses the naturally occurring anti-nutritional factors (ANF) in legumes and pulses, how processes like fermentation and germination impact nutrition and highlighting again that these ANF can enhance mycotoxin contamination.^133^^)^ Nutritional changes, coupled with the potential for mycotoxin contamination, underscore the importance of pulses as a food safety topic. The following sections will cover primary research on how the nutritional elements of pulses have changed when infected by various fungi.

### 3.1 Bean (*Phaseolus vulgaris* L.)

**Fig. 11.**
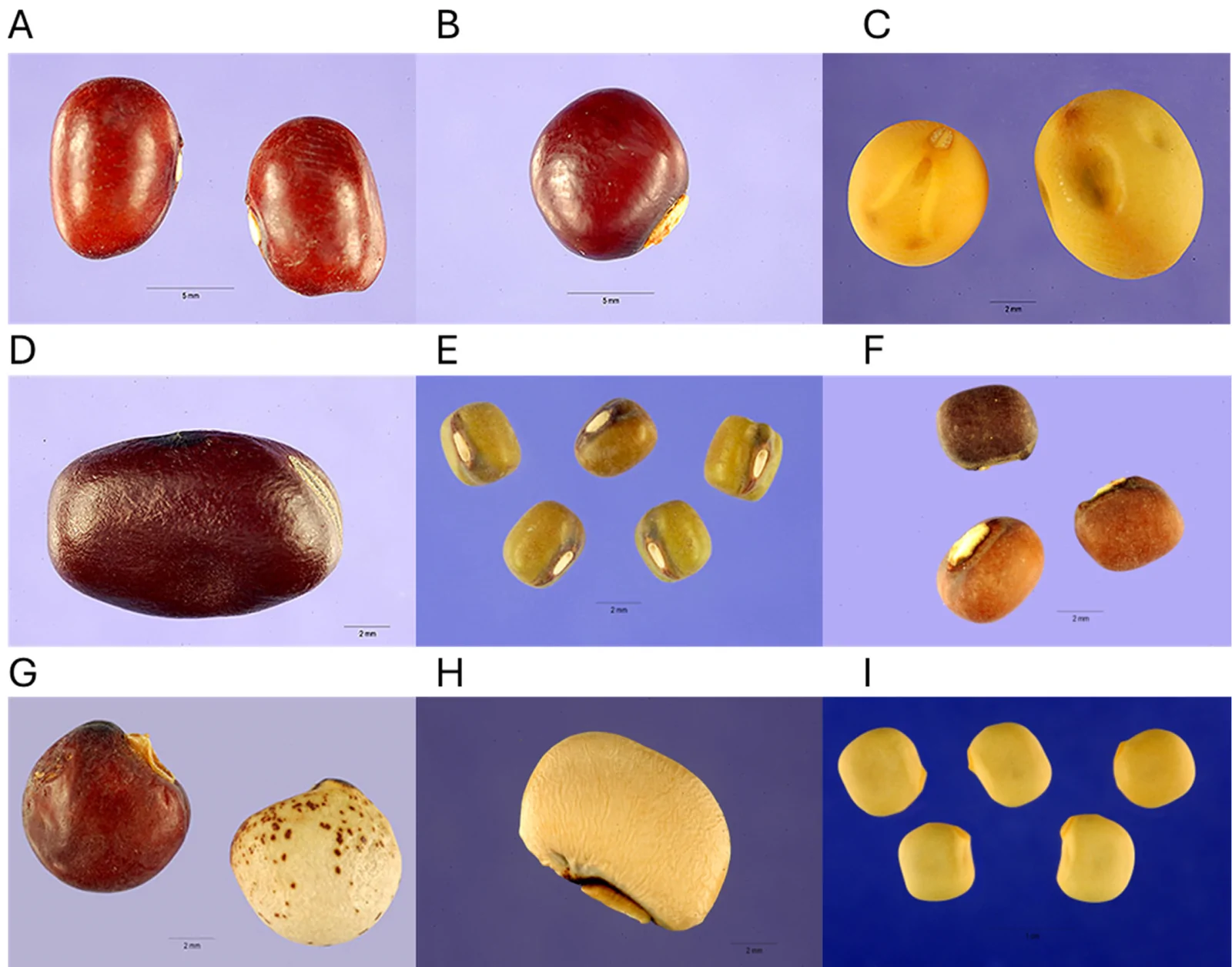
**Images of various pulses without infection. A)** Bean (*Phaseolus vulgaris* L.). **B)** Winged bean (*Psophocarpus tetragonolobus* (L.) DC.). **C)** Pea (*Pisum sativum* L.). **D)** Pigeon pea (*Cajanus cajan* (L.) Millsp.). **E)** Faba bean (*Vicia faba* L.). **F)** Mung bean (*Vigna radiata* (L.) R. Wilczek). **G)** Mungo bean (*Vigna mungo* (L.) Hepper). **H)** Cowpea (*Vigna unguiculata* (L.) Walp. ssp. *unguiculata*). **I)** Lupine (*Lupinus termis*). All images provided by the ARS Systematic Botany and Mycology Laboratory^181^^)^.

Beans are a staple across the world for their nutritional benefits (**Fig. 11A**). Beans are on average 25.9% protein, 4.3% fiber, 36.7% carbs and 1.3% fat^134^^)^. Dry beans were cultivated commercially in the US, including the states Colorado, Idaho, Michigan, Minnesota, Nebraska, North Dakota, and Washington^135^^)^. One and a half million acres of beans were planted and harvested in the US in 2024. The total production of beans in the US was 29.4 million hundredweights (CWT), and this accounts for $999 million in production.

Dry beans were also harvested in 44 other countries, including Brazil, Mexico and Kenya; these countries harvested over one million hectares (ha) of dry beans in 2022. Additionally, in Japan just over 29,000 ha of beans were harvested in 2022^135^^,^^136^^)^.

Our review identified studies that infected beans and compared nutritional characteristics. Beans were infected with *Aspergillus flavus*, *P. infestans, Aspergillus parasiticus*, *Aspergillus niger, Aspergillus terreus, Fusarium oxysporum*, and *F. verticillioides*, formerly known as *Fusarium moniliforme*^137^^,^^138^^,^^139^^,^^140^^,^^141^^,^^142^^)^. Across six studies that highlighted nutritional changes in beans due to fungal infection, there were clear trends. Across the studies, protein, fiber, lipids/fats, ash, carbohydrates and moisture almost always decreased. In cases where protein, ash, fiber or moisture increased during fungal infection, researchers speculated the increases could be due to how fungi convert carbohydrates to protein, or due to mycelial formation in the seeds^137^^)^. Additionally, to address differences between fungal species when examined, researchers suggest that fungi have different strategies for positioning and utilizing resources. All findings point to a decrease in the overall nutritive value of beans when infected with fungi. In order to preserve bean protein content, fungal infections of seeds should be avoided using appropriate measures^142^^)^.

Depending on the fungal infection, beans exhibited varying changes in protein content. Interestingly, infections with *Aspergillus flavus* showed conflicting results: one found an increase in protein, while the other observed a decrease in protein levels^137^^,^^138^^)^. These studies used the same method to measure protein, however, the seeds were steamed after infection when protein increased, whereas seeds were sterilized before infection in the study with decreased protein. Additionally, in studies investigating *A. parasiticus* infections, one study showed a large reduction in protein (>20%), whereas another showed a slight decrease in protein (<3%)^140^^,^^141^^)^. However, these discrepancies could be due to different lengths of incubation time.

Further investigations into bean protein levels and fungal infections are essential. Common beans can be infected by several fungal species, including *Rhizoctonia solani*, *Colletotrichum lindemuthianum*, *Sclerotinia sclerotiorum*, *Macrophomina phaseolina*, *Fusarium* spp., and *Cercospora* spp., as well as the others previously discussed^143^^)^. Overall, the findings indicate that beans are susceptible to fungal infection; therefore, it is important to establish safe guidance for harvesting and storage of beans.

### 3.2 Winged Bean (*Psophocarpus tetragonolobus* (L.) DC.)

Winged beans are a high nitrogen-fixing plant popularly grown in tropical regions (**Fig. 11B**). They are known for their high protein levels, as well as being rich in starch and B-complex vitamins. Winged beans are also great at tolerating drought, flooding, and extreme temperatures. Because of their hardiness, winged beans can be an asset to diversifying global food systems. However, a downside to winged beans is the presence of trypsin inhibitors that require the dried beans to be soaked, rinsed and cooked thoroughly before consumption^144^^)^.

For our review we identified one study that included winged bean^137^^)^. Decreases in lipid, ash, and carbohydrate were seen, while protein and fiber increased. Further research is needed to increase confidence and understanding of these findings in winged beans. Currently, winged beans face false rust and leaf spot pressure, but with introduction to other climatic regions, new fungal infections could emerge^144^^)^.

### 3.3 Pea (*Pisum sativum* L.)

Peas are a popular crop grown in many countries as it increases soil nutrients and decreases disease incidence (**Fig. 11C**)^1^^)^. Dry peas have an average of 23.5% protein, 65% carbs, and 25.5% fiber^5^^)^. They are becoming a major benchmark protein due to their low cost and high functionality^1^^)^. Dry peas were most harvested in the Russian Federation, Canada and India in 2022. Japan’s imputed dry pea harvest was 455 ha in 2022^136^^)^. Additionally, in the US, peas were harvested in Idaho, Montana, Nebraska, North Dakota and Washington in 2024. A total of 988,000 acres were planted, with 947,000 acres harvested across the US in 2024. The total production of peas in 2024 was 19 million CWT, which translates to a total of $275 million of dry peas produced^135^^)^.

While peas are becoming a more popular protein additive to food products, we identified two studies concerning pea nutritional quality and fungal infections of *Aspergillus parasiticus* and *F. verticillioides*^140^^,^^141^^)^. Both studies showed decreases across pea protein, lipid/fat, and fiber (if collected) and an increase in moisture. Contrastingly, one study found an increase in ash while another had a decrease.

While the findings of these studies varied depending on the type of fungal infection, pea nutritional components were affected. These findings highlight that different fungal infections have distinct impacts on pea seeds. Further research studying the mechanisms of fungal pea infection and subsequent mycotoxin accumulation is necessary.

### 3.4 Pigeon Pea (*Cajanus cajan* (L.) Huth)

Pigeon peas are a nitrogen-fixing and drought-tolerant plant protein cultivated across Africa and India for thousands of years (**Fig. 11D**)^1^^,^^145^^)^. Today, they are cultivated across Asia, Africa and South America^1^^)^. The highest harvest of pigeon peas (in terms of area harvested) was in India and Kenya in 2022^136^^)^. This plant protein is notably high in some limiting amino acids, such as methionine, lysine, and tryptophan. It is also high in vitamins and minerals, including B vitamins, magnesium, potassium, phosphorus, copper, manganese, calcium and choline. On average, pigeon peas contain 15-29% protein^1^^)^. Although this plant protein grows best in the tropics and subtropics, pigeon peas have been adapted to grow in the Southern US, Hawaii and Puerto Rico^145^^)^.

For our review, we identified studies highlighting pigeon pea nutritional changes due to infections with *Aspergillus flavus*, *Aspergillus fumigatus*, *Aspergillus niger*, *C. spicifera*, *F. verticillioides* and *R. stolonifer*^146^^,^^147^^)^. Both studies reported a marked decrease in protein levels when infected with fungi. Overall, pigeon pea composition changes caused by fungi are understudied. With pigeon peas being a staple across many tropical and subtropical areas, increased understanding of how fungal disease and mycotoxins impact pigeon peas should be a focus of future studies. Additionally, pigeon pea plants have also been found to be susceptible to many insects and *Fusarium* wilt^145^^)^. It would be valuable to explore how mycotoxins, specifically AF and FUM, impact pigeon pea production, harvest, and consumption.

### 3.5 Faba Bean (*Vicia faba* L.)

Faba beans, also commonly referred to as broad beans, horse beans, haba beans, or fava beans, are grown in Western Europe, Australia, China, Ethiopia, Sudan, and Egypt (**Fig. 11E**)^1^^)^. Faba beans are mostly harvested in Australia, the UK and Morocco, at 287,500, 211,654 and 80,462 ha, respectively. Japan harvested an estimated 96 ha of faba beans in 2022^136^^)^. Faba beans are common across the Middle East and have been cultivated there for thousands of years. These plants grow best in temperate and subtropical areas, as well as high elevations in the tropics^148^^)^. The faba bean plant is known for its hardiness, growing in cold, high salinity climates, and flourishing in clay soils^1^^)^. One drawback to consuming faba beans is that some individuals with the specific genetic variant can develop favism, causing acute hemolytic anemia. Faba beans have approximately 24% protein and 50% carbohydrates. These beans are also high in the amino acid, L-DOPA, used in treatment of Parkinson’s Disease and dopamine-responsive dystonia^148^^)^.

Faba beans were found to have an increase in protein when infected with *Aspergillus flavus* and decreases in carbohydrates, fiber and lipids^149^^)^. However, a larger sample size and further study is needed to validate these results. Additionally, the classification method of diseased vs. healthy seeds based on discoloration is not a useful method for determining fungal infection^150^^)^. A potential downside to increased protein content following *Aspergillus flavus* infection is AFB_1_ was found at levels of 85 ppb in faba beans after 30 days of exposure. Interestingly, Aziz and Mahrous discovered that irradiating the seeds prior to infection decreased AFB_1_ levels in faba beans, but it also decreased protein and carbohydrate levels substantially^149^^)^. Therefore, irradiation would not be a viable option for farmers aiming to maximize nutritional value of their faba bean crops.

One solution to combat disease in faba beans is to use a diverse rotational cropping system. This method has been shown to help break disease cycles of *Gaeumannomyces graminis* (take-all) in cereals and *Sclerotinia minor* (lettuce drop) in lettuces. A similar study could be conducted to investigate other common fungal infections, such as *Aspergillus* and *Fusarium* spp., as faba beans are largely susceptible to aphids and fungal diseases infecting leaves and pods^148^^)^. It is important for food safety that more studies focus on faba bean susceptibility to fungal infections and the impacts of those infections on seed composition.

### 3.6 Mung Bean (*Vigna radiata* (L.) R Wilczek)

Mung beans are a major crop in East and Southeast Asia, as well as India (**Fig. 11F**). They are a low-cost raw material and have emerged as a plant-based protein source in the US^1^^)^. Mung bean is also commonly referred to as green gram, or golden gram, for its distinct yellow-greenish color. These beans are grown across the tropics and subtropics, and are also increasingly produced in Oklahoma in the US^151^^)^.

Mung beans were infected with *Aspergillus flavus*, *Aspergillus fumigatus*, *Aspergillus niger*, *C. spicifera*, *F. verticillioides*, *R. stolonifer*, *Alternaria alternata*, *F. oxysporum*, *M. phaseolina*, *Alternaria japonica* previously recognized as *Alternaria raphani*, and/or *C. kikuchii*^146^^,^^147^^,^^152^^)^. Mung bean protein was reduced across the studies but decreases varied depending on the species of fungi^146^^,^^147^^,^^152^^)^. This is important as mung beans are susceptible to many fungal infections, including white mold, *Rhizoctonia* spp., and mildew^151^^)^. As mung beans become more of a staple as a plant-based protein in the US and globally, continued research should be done to address their nutritional composition changes based on fungal infection and mycotoxins.

### 3.7 Mungo Bean (*Vigna mungo* (L.) Hepper)

Mungo bean, also known as black gram, urd bean, and urad bean, is widely grown in East Asia and India with adaptation to drier tropical regions (**Fig. 11G**)^153^^)^. Dried seeds are usually cooked or ground into flour and incorporated into papadum, idli and dosa in Indian cuisine^154^^)^. Mungo beans have a nutritional composition of 25.2% protein, 1.64% fat, 3.3% ash, 59% carbohydrate, and 18.3% fiber^155^^)^.

Across the studies we identified, mungo bean seeds stored with fungi had losses in protein and carbohydrate content with AF levels varying based on region (when measured). Mungo bean protein levels decreased at different rates depending on the fungal infection^146^^,^^156^^)^. However, more research is needed to have confidence in mungo bean nutritional composition changes as well as mycotoxin accumulation across infected samples.

### 3.8 Lentil (*Lens culinaris Medik. or Vicia lens (L.) Coss. & Germ.*)

Lentils are a hardy crop cultivated globally, thriving in semi-arid areas without irrigation.^157^^)^ As one of the oldest known crops, they are a staple in Indian cuisine.^1^^)^ In a recent review on lentils, mycotoxins and mycoflora infection are highly prevalent and need to be further addressed in pre- and post-harvest settings^158^^)^. Interestingly, the US, Canada and Australia export most of their lentil crop to Asian countries^157^^)^. The top lentil-growing countries were Canada, India, Australia, US and Turkey in 2022^1^^,^^136^^)^. In 2024, the US produced 9 million CWT of lentils, corresponding to just under $222 million in annual revenue. Across the US, roughly 936,000 acres of lentils were planted, and 900,000 acres of lentils were harvested^135^^)^. Lentils are an excellent source of protein and fiber, and are also high in lysine, folate, thiamin, phosphorus and iron. However, lentils are low in methionine and cysteine; when consumed with cereals, they form a complete protein^157^^)^. The average composition for lentils is 25.8% protein, 60% carbohydrates, and 30.5% fiber^5^^)^.

Lentils are susceptible to many insects and fungal infections, including root rots or wilts by *Rhizoctonia* and *Fusarium* spp., among others, and seedborne fungal infections^157^^)^. They are also sensitive to flooding, and this must be taken into consideration when planting^1^^)^. The findings identified indicate mixed results: one study’s infection with *A. flavus* measured an increase in protein and fiber, whereas the two other studies found decreases in protein levels and some other nutrients. Various species of fungi were examined, including *Aspergillus flavus*, *Aspergillus niger*, *Alternaria alternata*, *F. oxysporum*, *M. phaseolina*, *F. verticillioides*, *Alternaria raphani*, or *C. kikuchii* and the changes in nutrient levels noted varied depending on the infection^137^^,^^147^^,^^152^^)^. More research to confirm these results and characterize potential mycotoxin production should be addressed in future studies.

### 3.9 Cowpea (*Vigna* spp.)

Cowpeas, also known as black-eyed peas or yardlong beans, are cultivated across the southern US, Middle East, Africa, Asia, tropics and subtropics (**Fig. 11H**)^159^^)^. They are well-adapted to the humidity of the tropics and temperate zones and are drought resistant. Cowpea is sometimes grown as a green manure crop, nitrogen-fixing crop, or to control erosion^160^^)^. Major producers of cowpea include Niger, Mali, Senegal and Kenya, with over 200 thousand ha harvested in 2022^136^^)^. The nutritional composition of cowpea is 24% protein, 53% carbohydrates and 2% fat. Cowpea is rich in lysine and tryptophan, but deficient in methionine and cystine. Cowpeas are critical to supplying adequate protein in India and East Asia, so crop harvest safety is important^159^^,^^160^^)^.

Cowpeas (*Vigna unguiculata* L. and *Vigna sinensis*) were infected with *Alternaria alternata*, *F. oxysporum*, *F. verticillioides*, *Alternaria raphani*, *C. kikuchii, F. oxysporum* f. sp. *tracheiphilum*, *Aspergillus flavus*, *Aspergillus niger*, and *M. phaseolina* across three separate studies^138^^,^^152^^,^^161^^)^. Trends included decreases in protein levels. Other nutritional elements measured in at least one of the studies showed decreases in sugar, carbohydrates, fat, fiber, ash and moisture.

Again, the magnitude of these decreases varied by fungal species. In addition to the fungal infections mentioned, cowpeas have been known to be susceptible to *Fusarium* wilt, root rot, damping off, and southern blight^159^^,^^160^^)^. Given that cowpeas are vulnerable to both pre- and post-harvest fungal infection, ensuring the safety of this food supply is crucial.

### 3.10 Lupine (*Lupinus* spp. L.)

Lupine, also known as lupin, is grown in Australia, Europe, Russia and the Americas (**Fig. 11I**). Depending on the variety of lupine, there are certain alkaloids present, lupanine and sparteine, that limit the use of lupine in food and feed products. In the 1920s, German plant breeders developed sweet lupine varieties with low or no alkaloids that do not need any extra processing before consumption. In Australia, the limit for alkaloid content is 0.02% for sweet lupines. Lupine has been cultivated for centuries across the Mediterranean region, most likely beginning in Egypt. In the US, lupine is usually utilized as flour due to its high protein content and a variety of macro and micronutrients. It can also be mixed with meat products to increase nutritional value and improve texture. Additionally, lupine contains 32-38% protein, 10% oil, and doesn’t have any trypsin inhibitors^121^^,^^162^^)^.

Lupine (*Lupinus termis*) was infected with *Aspergillus flavus* and *F. oxysporum* where protein, carbohydrate, fat, fiber, ash and moisture were measured. Protein showed decreases in both studies, however there were varying results for other components. For instance, ash, fiber and moisture increased in one study but decreased in another study^138^^,^^141^^)^. In these studies, lupine nutritional components showed varying results, which could be caused by experimental design. Overall, the infections with fungi cause a decrease in protein, carbohydrate and lipid levels^138^^,^^141^^)^. Other fungal pathogens, like *Diaporthe*, *Fusarium*, *Rhizoctonia*, *Phytophthora*, *Pythium*, *Ascochyta* and *Botrytis* spp. also cause disease on lupine plants^121^^,^^162^^)^. To date, characterizing resistance to fungi in lupine is in the early stages. A focus on genetic factors influencing lupine susceptibility could enable the development of lupine varieties that are resistant to fungal infections and capable of growing in wetter, higher pH soils^162^^,^^163^^)^.

### 3.11 Chickpea (*Cicer arietinum* L.)

Chickpeas, also known as garbanzo beans, are a major crop in India and an emerging protein source in the US.^1^^)^ They are grown across southern Europe, the Middle East, North Africa, Australia, China and the Americas^164^^)^. Top producers of chickpeas are India, Pakistan, and Australia with over 600 thousand ha harvested in 2022^136^^)^. In 2024, just over 500 thousand acres of chickpeas were planted and just under 500 thousand acres harvested in the US. This led to 6 million CWT in production, equating to $172 million in revenue^135^^)^. Chickpeas come in two varieties, Desi (microsperma) and Kabuli (macrosperma)^164^^)^. Chickpeas have a favorable composition, with an average of 22.4% protein, 57.8% carbohydrates, 5% fat, and 10.8% fiber^5^^,^^165^^)^. Chickpea protein could be a possible alternative to pea protein, which some people are hesitant to adopt due to allergen concerns^1^^)^. However, chickpeas are deficient in the amino acids’ methionine and cystine^165^^)^. Chickpeas have even greater potential as plant-based proteins if byproduct utilization of starch is improved^1^^)^.

We surveyed studies of chickpeas with varying fungal prevalence, including *Aspergillus flavus*, *Aspergillus fumigatus*, *Aspergillus niger*, *C. spicifera*, *F. verticillioides, R. stolonifer, Aspergillus oryzae*, *Aspergillus quericinus*, or *Aspergillus nidulans*^146^^,^^150^^,^^166^^,^^167^^)^. Across these studies, chickpea seeds had a reduction in protein, weight, carbohydrates and starch following infection^146^^,^^166^^)^. Interestingly, one study found differences in nutritional composition between Desi and Kabuli chickpea varieties after infection^166^^)^. More concerningly, one study found chickpeas being sold at markets in Pakistan had AF levels higher than the maximum limit set by the EU^167^^)^. Chickpea plants are known to be susceptible to Ascochyta blight, Rhizoctonia root rot, Pythium rot, and Fusarium wilt, in addition to the fungi mentioned above^165^^)^. Since chickpeas are a staple in many countries, more research on the nutritional composition of chickpea seeds after fungal infection and potential mycotoxin exposure is essential to ensure global food safety.

### 3.12 Broad Impacts and Key Findings

Across all pulse species described in this review, there are several clear patterns emerging regarding the fungi most frequently associated with infection and decreased nutritional value. The most recurrent and impactful pathogens across pulse types are species of *Aspergillus* and *Fusarium*, which appear in nearly every crop category reviewed. *Aspergillus* spp. are particularly consequential due to their global distribution, enzymatic activity, and capacity to produce AFs. Similarly, *F. oxysporum* and *F. verticillioides* are widespread seedborne pathogens that consistently reduce protein and carbohydrate content while increasing moisture. Other fungi, including *Alternaria*, *Macrophomina*, *Curvularia*, *Rhizopus*, and *Phytophthora* spp., appear less frequently in the literature on pulses, but still contribute to measurable nutrient losses, particularly in mung bean, lentil, and cowpea.

Despite differences in fungal species and pulse physiology, nutritional changes showed consistent trends across studies. Protein content typically decreased following infection. Some infections were exceptions, but likely reflect fungal biomass accumulation or concentration effects caused by the depletion of other nutrients rather than genuine improvements in nutritional quality. Carbohydrates and lipids almost always decreased, most likely due to fungal amylase and lipase activity^168^^)^. Ash and fiber show mixed responses, with some increases likely attributed to fungal cell wall material or concentration effects as other nutrients decline^140^^)^.

Across these studies, consistent gaps in the literature remain. These include the absence of standardized methods for quantifying infection severity and nutrient loss, limited data on amino acid profiles, and lack of coordinated global surveillance for mycotoxins in pulses. These gaps hinder direct comparison across studies and limit the development of targeted mitigation strategies. Although these gaps are large, several areas identified as priorities for future research have shown encouraging progress. As we have mentioned extensively throughout this review, there are many fruitful avenues for future research to focus on including: 1) researching resistance to mycotoxigenic fungi *in planta* or during harvest and storage, 2) developing a standardized and rapid method for assessing fungal infection and detecting nutritional composition, 3) conducting an in-depth look at the protein content and amino acid profiles of pulses before and after infection, 4) developing genetic resistance to emerging fungal infections, as pulses become more popular with farmers and consumers, 5) establishing global limits and guidance for major mycotoxins in pulses, like AF and FUM, and 6) developing strategies for reducing or eliminating fungal infection and mycotoxin contamination pre- and post-harvest.

Advances in rapid and standardized detection methods (item 2) are particularly notable. Near-infrared spectroscopy (NIRS) and hyperspectral imaging have been increasingly applied to pulses for non-destructive detection of fungal contamination and, in some cases, associated changes in nutrient composition^169^^,^^170^^)^. These optical methods have demonstrated high accuracy for detecting *Aspergillus* infection and AF contamination, and recent work has integrated machine-learning models to improve classification performance^171^^)^. Molecular approaches, including qPCR-based assays for *Aspergillus* and *Fusarium*, have also been adapted for pulse matrices and offer sensitive, species-specific detection^172^^,^^173^^)^. In parallel, portable biosensors for AF detection have become more robust and field-deployable, representing an important step toward harmonized, rapid screening tools suitable for both producers and regulators^174^^)^. While these technologies are not yet standardized globally, their development demonstrates meaningful progress toward the goals outlined in this review.

Progress has also been made toward establishing global limits and guidance for major mycotoxins in pulses (item 5). Although regulatory frameworks remain inconsistent across regions, several international bodies, including Codex Alimentarius, have begun evaluating the need for pulse-specific limits for AFs and FUM as pulse consumption and trade expand^19^^,^^175^^)^. China is notably one of the only countries to our knowledge with mycotoxin limits set for beans and bean products^33^^)^. Although comprehensive, pulse-specific regulations are still lacking, these developments represent meaningful progress toward establishing global guidance. Together, these advances underscore the increasing attention directed toward fungal contamination and mycotoxin risk in pulses.

## 4. Conclusion

Pulses are rapidly becoming a staple source of protein in the US as the market for plant-based proteins increases. Pulses are often harvested for dry seeds and flour. While nutritional composition changes due to fungal infections have been investigated, many pulses remain understudied. Additionally, mycotoxin contamination affects many plants, including the plant proteins we have discussed in this review. However, not all plant-based proteins are affected equally. Chickpeas are one of the most impacted pulses by AF, followed by beans, then lentils^19^^,^^176^^)^. FUM is the next most common toxin present in plant-based proteins^19^^)^. As pulse crops continue growing in popularity (see **Fig. 10**), it is important to address how nutritional composition and mycotoxin prevalence in pulses are impacted by different fungal infections. Continued work on resistance breeding, genetic approaches, nutrient profiling, and mitigation strategies will be essential to fully address the challenges identified in this review and to support the safe, nutritious expansion of pulse crops worldwide.

## 5. List of Abbreviations

AAL toxin -– *Alternaria alternata lycopersici* toxin

AF – aflatoxin

AFB_1_ -– aflatoxin B1

AFM_1_ – aflatoxin M1

AME – alternariol monomethyl ether

ANF – anti-nutritional factors

AOH – Alternariol alternariol

ATA – alimentary toxic aleukia

ATP – Adenosine triphosphate

bw/d – body weight per day

CONTAM – EFSA Panel on Contaminants in the Food Chain

CPA – cyclopiazonic acid

CWT – hundredweights

DON – deoxynivalenol

DRV – Daily Reference Value

EFSA – European Food Safety Authority

EU – European Union

FAMIC – Food and Agricultural Materials Inspection Center

FAO – Food and Agricultural Organization of the United Nations

FAOSTAT – Food and Agriculture Organization of the United Nations, Statistics Division

FDA – US Food and Drug Administration

FSCJ – Food Safety Commission of Japan

FSIS – Food Safety and Inspection Service

FUM – fumonisin

GFI – Good Food Institute

GRAS – generally recognized as safe

ha – hectares

IARC – International Agency for Research on Cancer

JECFA – Joint FAO/WHO Expert Committee on Food Additives

L-DOPA – L-3,4-dihydroxyphenylalanine, also known as levodopa

LEM – leukoencephalomalacia

ML – maximum levels

NIRS – Near-infrared spectroscopy

OTA – ochratoxin A

PA – puberulic acid

PES – pulmonary edema syndrome

PHO – phomopsins

PHO-A – phomopsin A

PHO-B – phomopsin B

PHO-C – phomopsin C

PHO-D – phomopsin D

PHO-E – phomopsin E

PMTDI – provisional maximum tolerable daily intake

ppb – parts per billion

ppm – parts per million

RNA – ribonucleic acid

ROS – reactive oxygen species

RYR – red yeast rice

spp. – species

TDI – tolerable daily intake

TeA – tenuazonic acid

UN – United Nations

US – United States

USDA – United States Department of Agriculture

USSR – Union of Soviet Socialist Republics

WHO – World Health Organization (United Nations)

ZEN – zearalenone
